# Gender, kinship, and other social predictors of incrimination in the inquisition register of Bologna (1291–1310): Results from an exponential random graph model

**DOI:** 10.1371/journal.pone.0315467

**Published:** 2025-02-11

**Authors:** David Zbíral, Katia Riccardo, Tomáš Hampejs, Zoltán Brys

**Affiliations:** Centre for the Digital Research of Religion, Department for the Study of Religions, Faculty of Arts, Masaryk University, Brno, Czech Republic; University of Glasgow, UNITED KINGDOM OF GREAT BRITAIN AND NORTHERN IRELAND

## Abstract

The medieval inquisition of heresy strongly relied on depositions, where witnesses were expected to report on the crimes of others and oneself. The resulting patterns of incrimination could be influenced by various factors, including the characteristics of the underlying dissident social network; the investigators’ choices and biases; the trial circumstances, some of which must have exerted considerable pressure upon deponents; and the deponents’ decisions to protect some suspects more than others. This case study aimed at disentangling selected social factors of incrimination in the register of the inquisition in Bologna, 1291–1310. We used social network analysis and, more specifically, an Exponential Random Graph Model (ERGM) to assess the influence of four social predictors: gender, churchperson status, membership of the urban “middle class”, and kinship ties between incriminators and the incriminated. To increase the validity of our results, we controlled for various trial circumstances and structural parameters of the incrimination network. Our model corroborated a tendency towards female-to-female incrimination, while we did not find any positive or negative tendency towards male-to-male incrimination. We identified no effect of churchperson status on incriminating, while we found that among Cathars, members of the middle class were more likely to be incriminated than people without this status. Our model also corroborated the tendency to incriminate one’s kinship group. Overall, our study underlines the relevance, but also the non-trivial operation, of social and demographic predictors in medieval heresy trials.

## Introduction

The cornerstone of medieval and early modern inquisition trials was the collection of incriminating testimonies against specific individuals. It is through incriminations elicited from people under interrogation–deponents–that inquisitors acquired leads for further investigation as well as evidence for concluding cases and passing sentences. If testimonies were to provide legally acceptable evidence, however, they had to be fairly specific concerning people, places, and circumstances. This makes inquisition records, as well as other trial records based on testimonies, valuable sources [1–5], especially regarding interactions within non-elite populations, which tend to be much less covered in other narrative or administrative sources [6].

Procedural needs explain very neatly the inquisitors’ focus on requesting and recording specific circumstantial evidence. What is, by contrast, largely implicit and more difficult to disentangle is the social conditioning of incriminations. We can certainly hypothesise that rather than providing a full and objective account of the involvement of their acquaintances, deponents would have been influenced by power relations (e.g., the potential costs of providing evidence against individuals with higher social capital), allegiances (e.g., the tendency to protect family members), and inquisitorial pressure (e.g., repeated interrogations; confrontation with information gathered from other testimonies; detention; much more rarely, torture) [7–10].

The importance of incrimination in pre-modern trials is broadly recognized and has been commented upon in the study of heresy inquisitions as well as in other contexts [8, 11, 12]. Most often, it has been studied as part of broader systems of social control [13–15], with which charges of heresy are inextricably intertwined [16, 17]. Bergemann [13, 18] has provided a compelling historical-sociological study of denunciations in early modern Spain, Romanov Russia, and Nazi Germany, comparing two models or regimes, the “coercive” versus the “voluntary” one, especially as regards differences in the number of incriminated persons, the nature of the transgression for which suspects were incriminated, and the social proximity between the incriminator and the incriminated (social relationship, same settlement, and same social status). Bergemann recognizes denunciation as “a social act, both at the dyadic level (…) and as a behavior embedded within a larger community and network of relationships” [13]. Without connection to Bergemann’s account of denunciations before the inquisition in early modern Spain, but in close conversation with research into the medieval inquisition of heresy, Rehr [19] pioneered the use of network analysis to study incriminations in medieval heresy trials, thus contributing to the nascent, yet already burgeoning scholarship either proposing to apply or actually applying network analysis to medieval inquisition material [4, 20–23]. Rehr examines whether a large-scale investigation of heresy in Lauragais (Languedoc, south-western France) in the 1240s targeted a specific social class, members of consular families, rather than evenly gathering and following the leads of investigation. Combining network centrality measures with source criticism, he concludes in favour of an anti-consular political agenda underpinning the campaign. Estévez et al. [10] examined an inquisition trial in Giaveno (1335) using dynamic network actor analysis to estimate three models, one for all denunciations, one for those targeting congregation fellows, and the last for those targeting kin. They conclude that women were more likely to denounce than men under the same pressure conditions, and that deponents had a tendency to conceal their congregation fellows and kinship group members and give their names only under pressure.

Considering how central the process of incriminating others was to inquisition trials, it is striking how little has been done in formal quantitative analysis of its social correlates. With the present case study of incrimination in medieval heresy trials, which fits into the broader framework of historical network research [24–28] and specifically network research into historical archival documents [29, 30], we want to help fill this gap by analysing an original dataset collected from the register of the inquisition of Bologna, 1291–1310 [31]. The dataset includes information on all other-people incriminations in the register, the relevant trial circumstances, and trial subjects (deponents and suspects). We use an Exponential Random Graph Model (ERGM) to assess the effects of trial subjects’ socio-demographic characteristics and social relations on the global patterns of incrimination in the register. We look specifically at gender, churchperson status, membership of the urban middle class, and kinship.

First, gender constitutes a factor which could have influenced incrimination in medieval heresy inquisitions. While there are probably few grounds to expect an intrinsic influence, we can posit at least three potential influences of gender on the resulting patterns of incrimination. Firstly, when deciding about what names to give, deponents would have considered the potential social costs of incriminating somebody of higher social status, and as part of such power relations [32, 33], women might have had to consider higher risks in incriminating men than the other way around. Secondly, any tendency towards same-gender interaction (gender homophily) in the underlying social network [34, 35] might well be reflected in incriminations. Thirdly, in spite of legal theory behind heresy trials which, unlike in some other legal contexts [36], considered men and women to be equal as witnesses, investigators could have been influenced by an implicit assumption about gender roles, resulting in male and female trial subjects being treated differently–for instance, investigating more thoroughly the crimes of men [37–41], giving more credence to male testimonies, and/or using female witnesses predominantly in specialised ways–for instance, to acquire knowledge about micro-communities less accessible through male testimonies.

As a second social predictor, we considered churchperson status; that is, the status of monk or friar, nun, or priest (of any rank). According to our expectations, people dependent on the Church for their subsistence and social standing might have been expected to display a higher level of collaboration with papally appointed inquisitors of heresy, and thus be more prone to give names. On the other hand, their close relations with the institutional Church might have limited their access to relevant information. It is thus important to assess whether churchperson status shows any effect on incriminating others, and in which direction it runs.

Third, social class, specifically the membership of the urban middle class, could have played a major role. Historians of medieval Christian nonconformism have remarked on a potential link between dissidence and some occupations promoted by the rising urbanisation in the 12th and 13th century, especially qualified craftsmen, moneylenders, and merchants, all of which we subsumed under the urban middle class. For instance, Paolini [42] concludes that after household and family, occupational contacts offered the third most important space for the diffusion of Cathar dissidence in Bologna. In another study, he perceives Catharism in Bologna as mostly supported by the urban middle class, especially well-off craftsmen [43]. Giansante [44, 45] has shown how moneylending was closely associated with dissidence and prone to raise the interest of inquisition tribunals. In addition, the urban middle class played an increasing role in various areas of social life. Galletti [46] shows how, in the second half of the 13th century, guilds acquired a key role in Bologna, with their members becoming an institutional part of the city council and active protagonists in the political and economic life of the city. These associations between dissidence and social class, which have been identified in literature specifically with regard to Cathar dissidence in Bologna at the turn of the 14th century, highlight the need to examine whether members of the middle class were prominent among suspects, be it because they were especially attracted to dissidence, or because Bologna inquisitors were biased towards targeting them in a situation similar to that described by Rehr [19], where consular families seem to have been special targets of a broad Languedocian inquisitorial campaign in the mid-1240s.

Lastly, kinship constitutes another social factor whose role in incriminating others in medieval heresy trials needs to be considered. The importance of kinship in both the transmission and everyday practice of medieval religious dissidence has been amply underlined [47, 48], and scholars have long observed that deponents in some inquisitions did not fail to incriminate their kinship group members [49]. On the other hand, the protection of kinship group members should result in the underrepresentation of in-family incriminations [35], because of emotional bonds and also pragmatic considerations relating to the confiscation of property [50, 51], the exclusion of convicted heretics and their descendants from public offices, and other social repercussions. We thus need to systematically consider kinship ties in incriminations.

Overall, therefore, the objective of this study was to better understand some of the social forces behind incriminations in pre-modern heresy trials. By collecting and examining the data on incriminations and trial subjects in the register of the inquisition of Bologna, 1291–1310, and constructing a statistical model, we were able to examine the effect of gender homophily between the incriminators and incriminated, the effect of churchperson status on giving names, the propensity of the urban middle class involved in Cathar dissidence to receive incriminations, and the role played by kinship.

## Materials and methods

### Study design

This is a historical case study investigating the long-term aggregated characteristics of individual behaviour through social network analysis. The examined network is a cross-sectional representation of incriminations–the relational acts of individual trial subjects at multiple time points within a behavioural space institutionally delimited by the operation of the inquisition tribunal in Bologna. The focus of our study was not individual decisions, but the long-term consistency of individual behaviours stipulated and constrained by institutional and other social conditions within a specific historical context. We considered incriminations not as transient events but as state-like events, due to their virtual irrevocability. The unit of analysis was the trial subject, defined as a person who was either a source or target of an incrimination in the register.

We collected data from the register, focusing on depositions, persons, and their incriminatory acts. For any deposition, we recorded the identifiers of the deponent, the inquisitor, and all the persons incriminated in this deposition. For any trial subject, we collected their name, characteristics, and relations covered by the register. We then transformed the data on depositions into a table of person-to-person edges, and explored them as a directed graph. Subsequently, we analysed the graph through an Exponential Random Graph Model (ERGM) to assess the effects of the chosen social predictors on the incriminations documented by the register, while controlling for several trial circumstances and topological parameters.

### Data source

Our data source was a complete edition of the Bologna inquisition register [52]. The register consists of 922 Latin documents dated from 1291 to 1310. It thus covers a still early, but already fully-matured phase of the papal inquisition of heresy, which was established in 1231 under the pontificate of Gregory IX. Among these 922 documents, 609 are depositions, that is interrogation records which typically yield incriminations. The dates of depositions range from 29 May 1291 to 23 July 1307.

For the most part, the register concerns two distinct dissident religious cultures of North-Central Italy at that time, known under the names of Cathars and Apostles. A smaller portion of the documents concerns more isolated cases of other heterodoxy. In addition to them, an extensive part of the register maps avowals of participation in anti-inquisitorial riots and the subsequent absolutions of the participants, some of whom had to pay a fine. Anti-Dominican and specifically anti-inquisitorial sentiments seem to have been widespread in Bologna at that time, even if they did not take the form of violent uprising. Nevertheless, they included an actual tumult on 13 May 1299, in which dozens of citizens expressed verbally their antipathies towards the inquisitors and/or sympathies towards religious nonconformists burned at the stake.

The Bologna inquisition trials were led by different inquisitors, sometimes assisted by co-inquisitors. The inquisitors who led interrogations which resulted in incriminations were Florius of Vicenza (29 May 1291 and 14 June 1291), Guido of Vicenza (17 July 1296 to 13 December 1303), Guido of Parma (19 May 1304 to 21 July 1305), and Bonifacius of Ferrara (4 July 1307 to 23 July 1307). The first three operated within the city of Bologna; Bonifacius of Ferrara was inquisitor in Modena but took charge of a series of Bologna-related trials.

Concerning the register, Dupré Theseider hypothesised that the majority of documents in it were medieval copies rather than originals [53], but the analysis of the notaries’ autographs and of the formal structure shows that the register actually consists of originals [52]. The text itself contains a handful of references to individual documents now lost, and some trials that lack final sentences. However, unlike what we find in various other medieval inquisition registers [54, 55], there is no trace of major losses or the existence of another register used by the tribunal in parallel during the same period. We can thus consider the register to give a reasonably comprehensive image of the inquisition in Bologna at the turn of the 14th century.

### Data collection and transformation for the analysis

The data collection was manual. A historian proficient in Latin–the second author–read closely the edition of the Latin text and collected information on all depositions and people involved in them into a set of spreadsheets. This information was then transformed into two data tables: (1) a table of nodes (trial subjects), including their sociodemographic characteristics and trial circumstances, which we treated as nodal attributes; and (2) a table of edges (incriminations).

#### Unit of analysis (trial subjects)

In the table of nodes, we included all trial subjects in the register of Bologna. We defined a trial subject as any individual person who was, in this register, either a deponent (i.e., gave a deposition in front of one of the inquisitors) or a suspect (i.e., was named by a deponent in front of an inquisitor in an incriminating context) or both.

While sometimes groups, such as families, were incriminated in passages which did not enable the identification of specific individuals who constituted the group, we did not include groups as nodes, as they did not meet our criteria for nodes. Furthermore, they would not perfectly match the set of variables we wanted to examine. The impact of not including groups should not be excessive, however, since the incrimination of groups whose specific individual members were not identifiable is a rare occurrence in this register: inquisitors followed a legal theory which led them to disentangle as much as possible the transgressions of specific named persons, because they needed to establish individual responsibility and assign individual punishment.

The process of compiling the table of nodes involved identifying persons on the basis of their names mentioned in the register. If there was any ambiguity, we cross-checked the identification of each person by looking at their attributes and relations (especially kinship ties). Almost all trial subjects could be unambiguously identified. Only eleven among them were incriminated but unnamed and only identified by their description, title or relation (e.g., “*homo brunus de Sancta Agata*”, “*homo tonsus*”, “*soror heremita de Plumatio*”, “*quidam sacerdos*”, “*uxor Bartholini Balugani*”). We also included these as nodes. There is a small probability that some of them might be identical to persons mentioned by name elsewhere in the register, but given their low number and negligible number of connections (1–2 in all cases), any overlap with named persons should not distort our picture of the social predictors of incriminations derived from the register.

#### Input nodal attributes

As input variables, we extracted the following socio-demographic attributes concerning persons:

1. *Gender* was coded as a binary variable. We established male or female gender on the basis of the first name. All first names in the register allowed us to make a decision. In addition, wherever possible, we cross-validated this datum through pronouns used, kinship relations (e.g., wife, brother, etc.) and/or honorary titles (lord, lady, etc.).
2. *Churchperson status* was coded as a binary variable. We considered as churchpersons: ordained priests, members of religious orders (for whom, in this kind of text, the order, the title “brother” or “sister”, and/or their home monastery would always be mentioned, e.g., in the form: “*Frater Anthonius (*…*) ordinis predicatorum*”, i.e. “brother Anthony from the Order of Preachers”), and holders of offices requiring churchperson status (e.g., “*rector ecclesie Sancti Benedicti*”, i.e. “parish priest of the church of Saint Benedict”). We delimited the *churchperson* category in such a way that it captured whether the person predominantly gained their subsistence on the basis of their office or status in the Church. Following this definition, we also included the *conversi*–that is religious who worked for a monastery but had not taken monastic vows.
3. *Middle class status* was coded as a binary variable. We established middle class status on the basis of occupation. We considered the following to be of middle class status: (a) merchants; (b) moneylenders; (c) free professions (teachers, students, lawyers, judges, notaries, scribes, and physicians); (d) craftsmen in well-respected trades (under this category, we included those featuring among the guilds–*società delle arti*–in the *Liber matricularum artium* of the years 1294–1321, preserved in the Archivio di Stato di Bologna: ASBo, Capitano del popolo, Liber matricularum artium 1294–1321, da c. CCXXV a c. CCXXXVIII [56]); and (e) spouses of any of these.
4. *Kinship group ID* was defined as a categorical variable. For every trial subject we established a kinship group ID based on the following process: if somewhere in the register someone mentioned that two trial subjects were in a close kinship relation (which we defined as spouse, sibling, parent, child, uncle/aunt, nephew/niece, grandparent, grandchild, parent-in-law, sibling-in-law, and child-in-law), we created a kinship tie between them. From the list of such kinship ties, we created an auxiliary graph, composed of kinship group components, and recorded the component ID as a nodal attribute. Each kinship group ID thus represents a fully connected component.

While some of these attributes are generally subject to change (e.g., a person can marry or remarry), during our data collection we observed no change of attribute value which could affect our analysis. There was thus no need to consider the change of attributes in the analysis.

#### Control nodal attributes

As control variables, we extracted ten binary flag attribute variables describing trial circumstances and three binary variables of personal affiliation to dissidence for each trial subject. As one person could have been interrogated multiple times and each of these interrogations could have had different circumstances, we aggregated the circumstance occurrences in nodal attributes describing whether the given trial subject was at least once subjected to a particular circumstance in the register.

1. *Deponent* expresses whether at least one deposition record of a given trial subject survives in the register. A deposition record is a type of document recording a testimony given by somebody. In this register, the deponent was always physically present in front of an inquisitor or his assistant, and gave testimony usually in oral form, which was recorded in writing by the notary.
2. *Redeponent* expresses whether there is more than one deposition record of a given trial subject extant in the register.
3. *Ever summoned* expresses whether the trial subject ever received a formal summons. We established the value on the basis of either the presence of a summoning letter in the register, or the mention of a summons in any of this trial subject’s depositions (worded for example as “*citatus comparuit*”, i.e. “appeared in court after being summoned”; “*citatus venit*”; “s/he came after being summoned”), or previous trial documentation which alludes to the summons. Conversely, in many cases, explicit mention is made of appearing in front of the inquisitor without a previous summons (“*sponte conparuit*”, i.e. “appeared in court spontaneously”). Where there is neither a mention of a summons or of the absence of a summons, we imputed zeros based on the assumption that the notaries would not fail to record information on a summons in one of these forms. Failing to report one’s own or other people’s crimes related to heresy voluntarily, even before being summoned, was in itself a punishable offence, and notaries would thus record a summons not only for the formal completeness of the record but also because it potentially constituted an aggravating circumstance in and of itself.
4. *Ever pledged* expresses whether for this trial subject the register records at least once a formal pledge–that is, a declaration of personal and financial liability for the truthfulness and completeness of one’s testimony (e.g., “*sub pena prestiti iuramenti et decem librarum bononinorum*”, i.e. “under oath and with the liability to pay ten Bolognese pounds”).
5. *Ever incarcerated* expresses whether the trial subject was ever held in investigative custody as part of the trials covered by the register. We did not include incarceration as a punishment, only investigative custody before a sentence.
6. *Ever tortured* expresses whether the trial subject was ever exposed to torture as part of the trials covered by the register.
7. Involved as a trial subject in interrogations led by *Florius of Vicenza*.
8. Involved as a trial subject in interrogations led by *Guido of Vicenza*.
9. Involved as a trial subject in interrogations led by *Guido of Parma*.
10. Involved as a trial subject in interrogations led by *Bonifacius of Ferrara*.
11. *Cathar-affiliated* was coded as a binary variable. We considered Cathar-affiliated those who were reported to have been involved in meetings involving baptism by the laying-on of hands (*consolamentum*) or the ritual greeting of those who had received this baptism (*reverencia*, in verbal form also *adoravit*), or were termed to be believers, supporters, defenders or receivers of people so baptised (full members).
12. *Apostle-affiliated* was coded as a binary variable. We considered Apostle-affiliated those who were termed “*apostoli*” (“Apostles”), “*de secta apostolorum*” (“from the sect of the Apostles”), “*pauperes*” (“poor ones”), “*minimi*” (“smallest ones”) or “*de secta et societate Gerardi Segarelli*, *Dolcini de Novaria et suorum sequacium*” (“from the sect and society of Gerard Segarelli, Dolcino of Novara and their followers”). In addition, we also counted those who engaged in meeting or assisting religious specialists so named.
13. *Other-heterodoxy-affiliated* was coded as a binary variable. It flags those who did not belong to a two-tier structure of full members vs. supporters, characteristic of Cathars and Apostles, but who reportedly engaged in unorthodox ideas or behaviours, or expressed more widespread discontent concerning the corrupted state of the Church or its religious orders. A varied set of people fall under this category: for instance, there are low prelates denounced by their fellow brothers for not observing Catholic practices, more pronounced religious sceptics, as well as persons strongly adverse to the Pope or the inquisition.

Alongside ID, we also included the name of the trial subject. This string variable was not directly involved in our analysis, but may be useful to scholars working with our dataset.

#### Outcome object (incrimination ties)

The outcome object in this study was all incriminations between trial subjects–a directed graph object encoded as a two-column data table, the first column representing the source node ID (incriminator) and the second column representing the target node ID (incriminated). We treated incrimination of one individual by another as a state rather than an event, especially since incrimination could not be undone: the social consequences of becoming a suspect in a heresy trial were already entailed. Accordingly, we did not consider multiple incriminations of one node by another (in the same or another deposition) as another tie, because it did not substantially contribute to the nature of the relation between them, the first as incriminator and the other as incriminated, let alone in any linear way. We did not include self-incriminations (i.e., where a deponent confessed their own guilt).

Self-incriminations apart, we considered as incriminations any mentions which identified a specific person as a participant in an action or belief which potentially constituted a criminal offence in a heresy trial. For instance, we did not include among incriminations mentions of family members for identification purposes, or denials (e.g., the deponent’s answer to the inquisitor that they knew nothing about the involvement of a specific person in heresy).

### Statistical analysis

#### Data preprocessing

*Imputation*. In the case of two input variables, churchperson status and middle class status, and in the case of all the control variables, we assumed that if a person had such a status, the information leading to the assignment of the flag would not be left unrecorded in the source. Concerning churchpersons, their status was considered to be so much a part of their identification (usually including an honorific, such as “sister”) that it would hardly be missed by the Bologna notaries. In the case of middle class status, the source certainly does not record everybody’s occupation; in particular, it entirely misses agricultural professions, which the Bologna notaries must have regarded more or less as default values. By contrast, however, they seem to minutely record the fine detail of urban occupations that we used to define middle class status, presumably both for identification purposes and as an outcome of the growing social significance of these professions in Bologna. This allowed us to impute zeros in cases where information on the occupation and/or office of both the trial subject and their spouse (if married) was missing.

*Manipulation of variables*. In order to avoid overgeneralization from a few cases, we decided to exclude binary nodal variables with fewer than 10 occurrences of either 0s or 1s. Two variables were excluded from the analysis: ever incarcerated (n = 4), and ever tortured (n = 3). Based on the topological properties of the network, we combined the four binary variables indicating trial subjects being investigated by each of the four investigators into a single binary variable (as described in the Descriptive Statistics section). Furthermore, to avoid collinearity, a Jaccard similarity measure was calculated among all pairs of nodal binary variables used by the ERGM (S1 Table), and where the value was above 0.65, we planned to exclude from the analysis that variable in any such pair which we considered less theoretically relevant [57]. However, only deponent status and ever summoned status exhibited high collinearity (SJ = 0.73), and since deponent status was only applied as a sampling space constraint, we did not remove any variable.

#### Network definition

A binary, directed graph of incriminations was defined based on the preprocessed attribute table and the relational incrimination table. “Binary” means that the incrimination tie does not have intensity, but is only either present or absent. In the context of this study, we treated even multiple incriminations going from one exact trial subject to another as simply the relation of incrimination (rather than, for example, turning it into weight). This is motivated by our conceptualisation of the resulting relation as a state. Nodes were defined as trial subjects (deponents or suspects), edges were defined as incrimination–specifically, the incrimination of somebody else.

#### Descriptive statistics

We report the descriptive statistics of the trial subjects’ categorical attributes using absolute and relative frequency. In the case of kinship group IDs, we report the frequency of those people who had no other kinship group members as we defined them among trial subjects.

For the descriptive statistics of the incrimination network, we calculated the following standard parameters: number of nodes, number of edges, number of mutual edges, median and interquartile range (IQR) of indegrees and outdegrees, reciprocity, reciprocity correlation coefficient [58], number of components, diameter of the largest component, and mean path length. We also calculated the indegree and outdegree frequencies to assess the variance of degree distributions. We compared the triad census of the observed graph and the median values of 10,000 random graphs of similar size. These values were informative for understanding the network topology, guiding the transformation and combination of existing variables and specifying the terms of the ERGM.

#### Exponential Random Graph Models

We analysed the graph using Exponential Random Graph Models (ERGMs). ERGMs are primarily used for the analysis of states, where the objective is to assess the aggregated outcome of a number of social predictors. Our use of ERGMs is justified by the fact that incriminations were virtually irrevocable and had a long-lasting effect, and that a new incrimination of the same suspect by the same deponent, either in the same or a subsequent deposition, did not add much to the essence of this relationship.

The use of ERGMs is not hindered by the longer time span covered by depositions in the register (17 years, 1291–1307). In fact, ERGMs are commonly used in analyses of the aggregated effects of long-term processes, provided that the network boundaries–delimited here not by actual dissidence, but by who incriminated whom in this particular register–are well defined.

ERGMs assume that the input terms are the tie-generation functions of the graph. They are used to calculate the conditional log-odd probabilities of having an edge between two random nodes for all the given input terms. Input terms can include topological (structural) terms, nodal attributes, and dyadic attributes. Beta coefficients of ERGM terms can be interpreted as in frequentist binary logistic regression: positive betas express an increased tendency, while negative betas express a decreased tendency [59].

Using the number of edges, the null model was first established as a reference for performance. Second, an ERGM model was built, whose terms contained selected topological terms (i.e., structural characteristics of the network), dyadic control terms, dyadic input terms, nodal-attribute-based control terms, and nodal-attribute-based input terms. We selected standard topological control terms (number of edges and degree heterogeneity) on the basis of descriptive network statistics. As input and control terms for the actual model, we included all the input and control variables described above which met our data coverage, variance and collinearity criteria. The separation between control and input terms was based on theory: selected social predictors of incrimination, the focus of our study, were included as input terms, while trial circumstances, from whose effects we wanted to isolate the effects of social predictors as much as possible, were included as control terms. Among dyadic control terms, we included reciprocity and religious affiliation homophily. Reciprocity is commonly observed in most social networks; religious affiliation homophily in incriminations is also evident as Cathars and Apostles were two religious cultures distinct from one another. Among dyadic input terms, we included kinship homophily and gender-based mixing patterns. Control nodal-attribute-based terms were trial circumstances. The input nodal-attribute-based terms were churchperson outdegree and middle class indegree. We studied the tendency of middle class people to have higher indegree separately for Cathars and Apostles. The expectations for each input term are outlined in Table 1.

**Table 1:** Expectations concerning the input ERGM terms.

Input ERGM term	Expectation
Kinship homophily	Positive tendency, as networks of religious dissidence in medieval Europe were kinship-based to a considerable degree, and given the pressure exerted upon them, deponents would not be likely to successfully hide their kin-related co-offenders from the inquisitors.
Tendency for female → female edges	Positive tendency, given the tendency to gender homophily in most human social networks.
Tendency for male → male edges	
Churchperson status tendency for higher outdegree	Positive tendency, as churchpersons would have been more likely to collaborate with inquisitors by giving names.
Middle class status tendency towards higher indegree among Cathars	Positive tendency, as the involvement of the middle class in Cathar dissidence was remarked in literature [43, 42].
Middle class status tendency towards higher indegree among Apostles	No tendency, as a similar affinity of the middle class to Apostolic dissidence was not observed, and this movement seems more rural than Cathar dissidence in Northcentral Italy.

To avoid multicollinearity, Variance Inflation Factor (VIF) values were calculated for all ERGM terms and those variables whose VIF was above 20 were excluded from the analysis [60]. We added one constraint to the ERGM graph-sampling space: we set the condition that non-deponents cannot have outdegrees, since by definition, only deponents can incriminate somebody. Accordingly, all nodal and dyadic ERGM terms were calculated for edges where the sender was a deponent. Average Marginal Effects (AME) were calculated to assess the relative importance of the input terms. To test the final ERGM, the Akaike Information Criterion (AIC) values for the null model and the complete model were compared. The ERGM was checked for convergence, and Markov chain Monte Carlo diagnostics and goodness-of-fit were applied to ensure that the coefficients could be interpreted. We only consider as significant those variables whose beta-p-values are less than 0.05.

#### Sensitivity analysis

To assess the stability of our ERGM results, we reran the model 100 times with 10% of randomly selected edges removed each time, and recalculated the ERGM beta values and p-values. We only considered such results as robust for which at least 90 of the 100 instances of the model resulted in p-values below 0.05.

#### Software

We performed all statistical calculations using the R programming language for statistical computing (R 4.3.1, Vienna, Austria). We calculated network measures using the following packages: igraph 1.5.1 [61], netUtils 0.8.2 [62], and netseq 1.0.2 [63]. For the ERGM, we used the ergm 4.5.0 package [64, 65]. We computed VIF and AME using ergMargins 0.1.3.1 [60, 66]. The R script is available in the Zenodo repository [31].

## Results

### Descriptive statistics

A majority (74.4%) of the 663 trial subjects were male, 14.5% of the trial subjects were churchpersons and 15.7% of them had an occupation fitting into the middle class status. Out of 541 kinship group IDs assigned, 56 (10.4%) had more than one kinship group member in the dataset, that is, formed an actual kinship group rather than an isolate in the kinship graph. Out of the 1074 incriminations, 46 (4.3%) occurred within a kinship group. Descriptive statistics of the trial subjects are available in Table 2. Due to their low variance, two variables were excluded from the analysis: ever incarcerated (n = 4) and ever tortured (n = 3).

**Table 2:** Characteristics of the trial subjects.

Variable	Trial subjects (n = 663)		
	Frequency	%	Binary variance
**INPUT VARIABLES**			
**Gender**			126.4
female	170	25.6	
male	493	74.4	
**Churchperson**			82.1
yes	96	14.5	
no	567	85.5	
**Middle class**			87.7
yes	104	15.7	
no	559	84.3	
Kinship group **ID**			130.2
No other kinship group member in the data	485	73.2	
At least one kinship group member in the data	178	26.8	
**Affiliation**			
Cathar-affiliated	129	19.5	103.9
Apostle-affiliated	305	46.0	164.7
Other-heterodoxy-affiliated	88	13.3	76.3
Not indicated	141	21.2	111.0
**CONTROL VARIABLES**			
**Deponent**			135.1
deponent	189	28.5	
non-deponent	474	71.5	
**Redeponent**			35.8
redeponent	38	5.7	
non-redeponent	625	94.3	
**Ever summoned**			122.9
summoned	163	24.6	
not summoned	500	75.4	
**Ever pledged**			34.0
pledged	36	5.4	
not pledged	627	94.6	
**Ever incarcerated**			4.0
incarcerated	4	0.6	
not incarcerated	659	99.4	
**Ever tortured**			3.0
tortured	3	0.5	
not tortured	660	99.5	
**Involved under** *			
Florius Vicenza	69	10.4	61.8
Guido Vicentinus	320	48.3	165.6
Guido Parmensis	298	44.9	164.1
Bonifacius of Ferrara	49	7.4	45.4

* can exceed 100% as one trial subject could be involved under multiple inquisitors.

**Fig 1:**
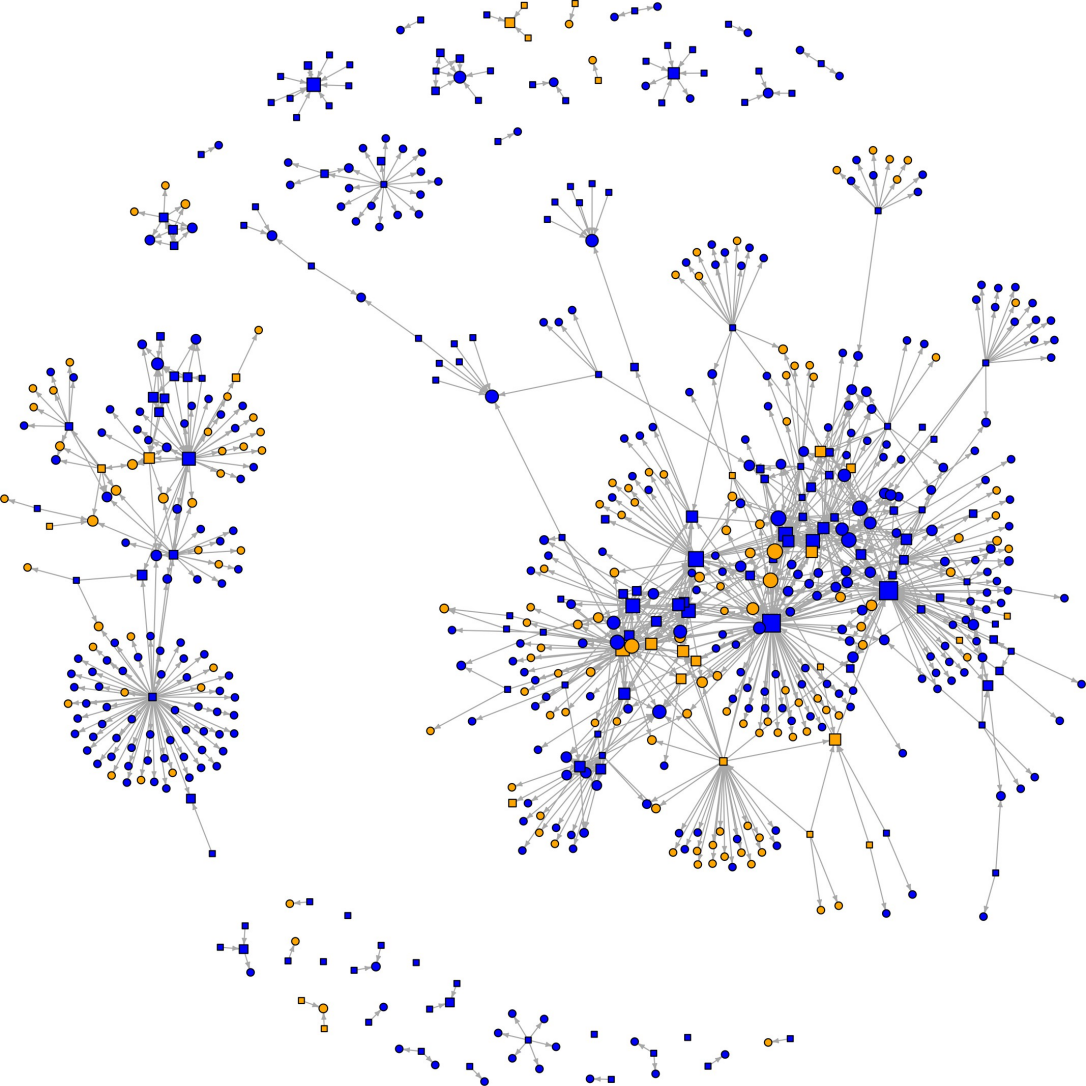
Fruchterman-Reingold visualisation of the Bologna incrimination network (663 nodes, 1074 ties). Each node represents a trial subject. Nodes are coloured according to their gender: blue colour represents males, orange colour represents females. The shape of the node represents the status of the trial subject in relation to deposition: square nodes represent deponents, circular nodes represent non-deponents. Nodes are scaled by the natural logarithm of their indegree.

The incrimination network consisted of 663 trial subjects and 1074 incriminations (Fig 1, Table 3).

**Table 3:** Characteristics of the directed incrimination network.

Parameters	Values (%) or [IQR]
**NODE CHARACTERISTICS**	
Number of nodes (trial subjects)	663
Number of isolated nodes	5 (0.75%)
**EDGE CHARACTERISTICS**	
Number of edges (representing incrimination of somebody else)	1074
Number of mutual edges	118 (10.99%)
**DEGREE CHARACTERISTICS**	
Average indegree and outdegree	1.62
Median [IQR] of indegrees	1 [1–2]
Median [IQR] of outdegrees	0 [0–1]
**TOPOLOGICAL CHARACTERISTICS**	
Density	0.0024
Reciprocity	0.11
Reciprocity correlation coefficient	0.11
Number of components	37
Diameter of the largest component	8
Mean path length	3.0

The indegree and outdegree counts are available as supplementary information (S2 and S3 Tables). Visual analysis indicated a difference between the outdegree distribution of trial subjects involved under the inquisitor Bonifacius of Ferrara or Florius de Vicenza and the outdegree distribution of trial subjects not involved under them (S1 Fig). Consequently, we controlled separately for their outdegree variance in the ERGM.

In the triad census (available in S4 Table), reciprocated edges (triad census state of 102) and mutual dyad with a transitive edge (triad census state of 111U) were overrepresented compared to the median values of 10,000 Erdős–Rényi random graphs of similar size. The overrepresentation of triad census state 111U (A↔B→C) might be the result of reciprocity, preferential attachment, and other outdegree-generation mechanisms in the network. These topological terms were included in the ERGM model as controls.

### ERGM analysis

As mentioned above, the ERGM configuration was constructed using topological control terms, dyadic control terms, dyadic input terms, nodal-attribute-based control terms and nodal-attribute-based input terms. Topological terms consisted of the number of edges, the tendency toward variance in outdegree (calculated separately for two subsets of nodes: for those who were involved under the inquisitor Bonifacius of Ferrara or Florius de Vicenza and for those who were not). Control dyadic variables were reciprocity and religious-affiliation-based homophily. Input dyadic variables were kinship homophily and gender-based mixing patterns. Control nodal attributes were the trial circumstances’ effects on outdegree. Input nodal attributes were the effect of middle class status on indegree (calculated separately for Cathars and Apostles) and the effect of churchperson status on outdegree. The sampling space was constrained to only those graphs where non-deponents had zero outdegree, since only deponents could send incrimination ties; in addition, nodal and dyadic ERGM terms were calculated for ties with deponent senders only.

The AIC value of the null model was -81,389, and the AIC of the full model was -169,960. The results of the ERGM model are available in Table 4. The results of standard Markov chain Monte Carlo diagnostics and the results of ERGM goodness-of-fit are available as supplementary information (S1, S2 Documents). The ERGM model converged and the diagnostics were acceptable.

**Table 4:** ERGM estimates.

ERGM term	N	B	std	p	VIF	AME
**Topological terms (control)**						
Number of edges	1074	-10.5	1.0	6.5E-25*	-	-
Tendency toward variance in the outdegree among trial subjects not involved under Bonifacius de Feraria or Florius Vicenza (α = 0.7)	240.6	-2.5	0.3	3.33E-16*	1.3	-0.003
Tendency toward variance in the outdegree among trial subjects involved under Bonifacius de Feraria or Florius de Vicenza (α = 0.7)	77.2	-0.7	0.3	3.89E-02*	1.19	-0.001
**Dyadic terms (control)**						
Reciprocity	118	2.2	0.1	1.30E-98*	1.1	0.003
Homophily among Cathars	1041	4.0	1.0	9.34E-05*	13.6	0.005
Homophily among Apostles	956	1.5	0.2	7.83E-16*	1.3	0.002
**Dyadic terms (input)**						
Kinship homophily	46	1.5	0.2	6.62E-11*	1.1	0.002
Tendency for female → female edges	81	1.0	0.3	4.35E-04*	2.0	0.001
Tendency for male → male edges	708	-0.1	0.2	6.16E-01	3.5	ns
**Nodal attribute based terms (control)**						
Repeated hearing outdegree	563	0.7	0.1	1.46E-08*	1.2	0.001
Ever summoned outdegree	936	0.5	0.2	3.49E-03*	1.1	0.001
Ever pledged outdegree	182	-0.3	0.2	8.01E-02	1.1	ns
**Nodal attribute based terms (input)**						
Gender outdegree	908	0.4	0.3	2.44E-01*	5.1	ns
Churchperson outdegree	202	-0.3	0.1	1.17E-02	1.1	<0.001
Middle class status indegree among Cathars	15	4.0	1.1	1.59E-04*	13.5	0.005
Middle class status indegree among Apostles	16	0.2	0.4	6.34E-01	1.2	ns

|V(G)| = 663, |E(G)| = 1074

AIC = -169,960 (null model AIC = -81,389)

N denotes the number of edges fitting the ERGM term, B denotes Beta, std denotes standard error, p denotes p-value, VIF denotes Variance Inflation Factor, AME denotes Average Marginal Effect (main effect). AME is only presented for significant effects (p-value < 0.05), * denotes significant effect, ns denotes non-significant effect, AIC denotes Akaike Information Criterion, |V(G)| denotes the number of nodes of the graph, |E(G)|denotes the number of edges of the graph. (AIC is negative due to the sampling-space constraints.)

### Sensitivity analysis

In accordance with the method described above, we randomly removed 10% of the edges and recalculated ERGM betas and p-values using the same ERGM sampling space and the same set of parameters. We repeated this process 100 times. During the repeated analysis, all ERGM models converged. The key results of the 100 models are available in Table 5.

**Table 5:** Results of the 100 ERGM models for the input ERGM terms.

Input ERGM term	Median beta [10th– 90th p]	90th percentile p-value
Kinship homophily	1.52 [1.42–1.60]	1.9E-09
Tendency for female → female edges	1.04 [0.93–1.20]	2.9E-03
Tendency for male → male edges	ns	9.1E-01
Churchperson status tendency for higher outdegree	ns	5.0E-02
Middle class status tendency for higher indegree among Cathars	3.93 [3.80–4.12]	5.8E-04
Middle class status tendency for higher indegree among Apostles	ns	8.7E-01

∀|V(G)| = 663, ∀|E(G)| = 967

Median AIC = -169,976, R(AIC) = -170,160 –-165,970

Each model was calculated with 10% of the edges randomly removed. B denotes Beta, p denotes p-value, AIC denotes Akaike Information Criterion, R denotes full range, and ns denotes non-significant effect expressing that the 90th percentile p-value was greater than 0.05.

Our sensitivity analysis confirmed the robustness of the following input terms: a positive tendency towards kinship homophily, female-to-female edges, and a higher indegree of middle-class-status trial subjects among Cathars (Table 5).

## Discussion

### Key results

In this study, we used Exponential Random Graph Models to evaluate the effect of four social predictors on incriminations recorded in the inquisition register of Bologna, 1291–1310: gender, churchperson status, middle class status, and kinship. Our results show that in the investigation covered by the Bologna register:

- Female deponents tended to incriminate other females, while no such gender homophily was observed among males
- The importance of members of the middle class among Cathars is highlighted by their tendency to be incriminated. A similar tendency was not confirmed for Apostles.
- Deponents tended to incriminate kinship group members more than people from outside their kinship group.
- Among trial circumstances, summonses and repeated interrogations tended to increase the overall number of names given by deponents ever subjected to those conditions. Pledges did not display a similar effect.

### Limitations

Most limitations of our study are related to the nature of our data collected from the inquisition register, others to our operationalizations and method.

Data availability limited our opportunities to investigate further predictors: we would have been interested in the effect of other socio-demographic factors, such as residence, age group, or socioeconomic status (of which our middle class status variable, based on a categorization of occupations, is only an approximation). However, the source offers only limited access to such information and no reliable opportunities for imputation. Similarly, while we are well informed about kinship, the register does not allow us to consider other potentially relevant social ties, since they are mentioned only rarely and haphazardly. Finally, we lack an independent account of the network of dissident relations and interactions to accurately evaluate the degree to which the effects unravelled by the model translate the characteristics of the underlying dissident network and to what degree they betray the influences of inquisitors’, notaries’, and deponents’ biases.

A second limitation is conditioned by various biases in inquisition records [2, 4, 34, 67, 68], which problematise the direct link between our data and the real milieu of religious dissidence. This limitation arguably affects more significantly studies which, unlike this one, investigate the actual values, behaviours, and illicit interactions of suspects, while here we used mostly the plain information on who incriminates whom, and types of information less likely to be affected by inquisitorial stereotypes and recording biases (occupation, churchperson status, kinship ties). However, we had to be careful in drawing conclusions as to the nature of the religious milieu from the patterns of incrimination.

A third limitation is that we had to set limits in terms of the detail of our data collection from this extensive source. We did not collect the specific transgressions for which trial subjects were incriminated, the mentioned alleviating or aggravating circumstances, and the characterisation of the pressure exerted by questions during interrogation as far as the extant record goes. A future study could certainly go further by collecting and categorising such circumstances, and using them as parameters in a more fine-grained explanatory model.

The fourth limitation consists in our operationalization of kinship group. The first aspect of our reduction is that we took into account only close kinship ties (as listed in the “Input nodal attributes” section), aiming at an optimal balance between theory (constituting the kinship graph from only kinship ties close enough to arguably matter in incriminations) and data coverage (choosing kinship ties systematically covered in inquisition documents, usually as part of the identification of persons, while more remote kinship and other social ties are not systematically recorded; thus, if we used them too, we would lose certainty in considering missing mentions of kinship anywhere in the register as signifying no kinship). The second aspect of our operationalization of kinship group is that from any connected component in the kinship graph constituted by such close kinship ties, we produced a fully connected kinship subgraph. This operationalization could be seen as too broad by some, because while only close kinship ties were used as paths to produce kinship groups, some nodes with the same kinship group ID could be somewhat distant from one another in kinship terms. By others, our operationalization could be seen as too restrictive, because there are other kinship ties which could have connected a node to a kinship subgraph, but we did not include them, since we thought the danger of them being left unrecorded was too great for us to consider the absence of data on those ties as the absence of a kinship tie. Thus, our definition of kinship group was based on specific choices, and our results are strictly limited to the framework thus defined, rather than directly related to the usually much broader emic definitions of family in medieval Europe.

The fifth limitation is related to the cross-sectional design of the study. While this design offers a logical way of providing a global view of the aggregated effects of the selected social predictors on incriminations, it focuses on the long-term sum of behaviours in the network rather than on factors affecting individual decision-making. Consequently, among control variables, we made an abstraction of the conditions of specific interrogations and treated trial circumstances as binary nodal attributes describing whether a given deponent was ever subjected to such circumstances.

### Interpretation

Generally, the model demonstrates the contributions of selected social predictors to the patterns of incrimination in the inquisition register of Bologna, 1291–1310. Of the four predictors considered, only churchperson status did not display a statistically significant effect, while gender, middle class status, and kinship all demonstrated a statistically significant influence on the network of incriminations we analysed. These influences held true in a model where we controlled for some potentially confounding factors, especially trial circumstances and selected topological parameters.

Our study unravelled intriguing patterns in gender combinations. We found a tendency among female deponents towards incriminating other females (while we found no such tendency among male deponents towards incriminating or refraining from incriminating male trial subjects). The female homophily in incriminations might be the result of two distinct influences: (1) a tendency to female homophily in the underlying dissident social network; and/or (2) the inquisitorial use of female deponents in a more specialised way; that is, to gain access specifically to female suspects. Our model does not allow us to disentangle which of these factors could have contributed more than the other to the observed pattern. The results do, however, provide a strong reason to consider the effects of gender homophily and other identity-related predictors on incriminations, not just in medieval inquisitions, but all the way up to recent and contemporary criminal cases, and not only on the side of investigating bodies, interrogators, and juries [69–71], but also on the side of the deponents themselves.

For churchperson deponents, we did not find a greater tendency to incriminate than for non-churchperson deponents. In other words, our model, based on data from the Bologna register, does not corroborate the notion that churchpersons had a special willingness to collaborate with the inquisition and/or any special (higher or lower) level of access to information about people suspected of heresy.

Passing on to the third social predictor examined, middle class status, our analysis of incriminations in the Bologna register corroborates the prominent role of middle class members as suspects in the Cathar milieu. Within this milieu, the holders of professions that we considered middle-class, such as moneylending, commerce, free professions, and crafts administered by guilds, were targeted more than nobles, servants, religious persons, craftsmen and service providers outside of guilds, and people with unrecorded occupations (many of whom would have been agricultural workers). Of course, here again the available data and the design of the model do not allow us to decide between alternative interpretations. The two obvious possibilities are that either middle class people actually did engage more in Cathar dissidence towards the end of the 13th century than other social classes, as some historiography would have it [42–44], or that the Bologna inquisition specifically targeted this social class–in a similar way, according to Rehr, that the large mid-1240s inquisition campaign in Languedoc targeted consular families [19]. Since we found no such tendency to higher indegree in the Apostolic milieu, however, we lean slightly more towards considering this a real social feature of Cathar dissidence covered by the register than arguing that the inquisitors were biased towards suspecting middle class people of heresy more.

Finally, our model shows a positive effect of kinship on incriminations: deponents covered by the Bologna register tended to incriminate kinship group members more than those with whom they did not have a kinship relation. Our model thus does not provide further evidence of kinship protection in incriminations, which was reported for another inquisition register [10]. Of course, this does not and cannot imply that kinship protection did not take place. Rather, the interpretation would be that whatever kinship protection was at play, it did not overpower the effect of kinship ties as a prominent channel in incriminations. Ultimately, it supports the understanding of kinship ties as playing a strong role in the underlying dissident network, a pattern which has constantly been underlined (even if rarely quantitatively expressed) in research about medieval Christian dissidence [47, 48]. Here, however, a critical consideration needs to be made, which puts this result in context with regard to descriptive statistics on the one hand and the functioning of ERGMs on the other. Descriptive statistics show that the network of incriminations in the Bologna register is, in fact, quite distinctly non-kinship-based. Only a tiny proportion of incrimination relations, 4.3%, actually occur within a kinship group, and in the auxiliary graph of kinship relations among trial subjects, there is a very high number of isolates (see Table 2)–that is, people without further kinship group members among trial subjects. This sparseness is probably not due to missing data; the Bologna inquisition notaries seemed just as interested in recording kinship ties of individual suspects as most other medieval inquisition notaries were. Rather, it points to the fact that the intensity of further investigation following kinship links was, in Bologna heresy inquisitions around 1300, not sufficient to systematically uncover more of the dissident milieu in which familial units were probably quite prominent [42]. For instance, while in some inquisitions, questions about the heresy of parents would systematically be asked [54], this was not the case here. The ERGM result is not “invalidated” by the descriptive results; rather, the contrast between the two points to an important feature of ERMGs. Even with this small proportion of incriminations following kinship ties, the model, trying to find the best fit for the input data provided, confirms that incriminations actually were more likely to occur within one’s family rather than at random. But if our model shows a positive effect of kinship on incriminations in spite of the small proportion of incriminations following kinship ties, it mostly points to the sheer extent of the knowledge we lack concerning other relevant spaces of social proximity which might have channelled religious dissidence in medieval Europe in equally important ways as kinship seems to have done.

### Generalisability and further directions

This article presents a case study of incriminations in a single inquisition register, albeit an extensive one compiled under the supervision of several inquisitors. Consequently, we would advise against extrapolation to incriminations in medieval heresy trials more broadly. Data from more inquisitorial investigations of heresy are needed before we attempt a synthetic view of the social factors influencing the patterns of incrimination in medieval heresy trials. Nevertheless, our study directs attention to specifically female homophily rather than gender homophily in general in incriminations (and possibly in dissidence); it provides the first corroboration through a statistical model of the importance of middle class suspects in the Cathar milieu (in contrast to the Apostolic milieu); and it argues for greater caution as to the interpretation of kinship’s role in incriminations and the underlying social circles of dissidence.

Among promising future directions, we propose comparative studies using one and the same model across different inquisition registers, thus putting our results into perspective and offering a more comprehensive insight into the deponents’ behaviour on the one hand and the inquisitors’ strategies of investigation on the other.

## Supporting information

S1 Table Jaccard similarity values of binary variables. (XLSX)

S2 Table Indegree distribution of the incrimination network. (XLSX)

S3 Table Outdegree distribution of the incrimination network. “Involved under” characteristics are not disjunct.
(XLSX)

S4 Table Triad census of the observed graph and the median values of 10,000 random graphs of similar size. (XLSX)

S1 Fig Visualization of outdegree distribution by “involved under” variables. Axes are logarithmic.
(TIFF)

S1 Document Markov chain Monte Carlo diagnostics of the main model. (PDF)

S2 Document Goodness of fit diagnostic of the main model. (PDF)
