# Supplementary figures and images for: Gender, kinship, and other social predictors of incrimination in the inquisition register of Bologna (1291–1310): Results from an exponential random graph model
Source: PLoS One. 2025 Feb 11;20(2):e0315467. doi: 10.1371/journal.pone.0315467 (PMC11813156; doi:10.1371/journal.pone.0315467)

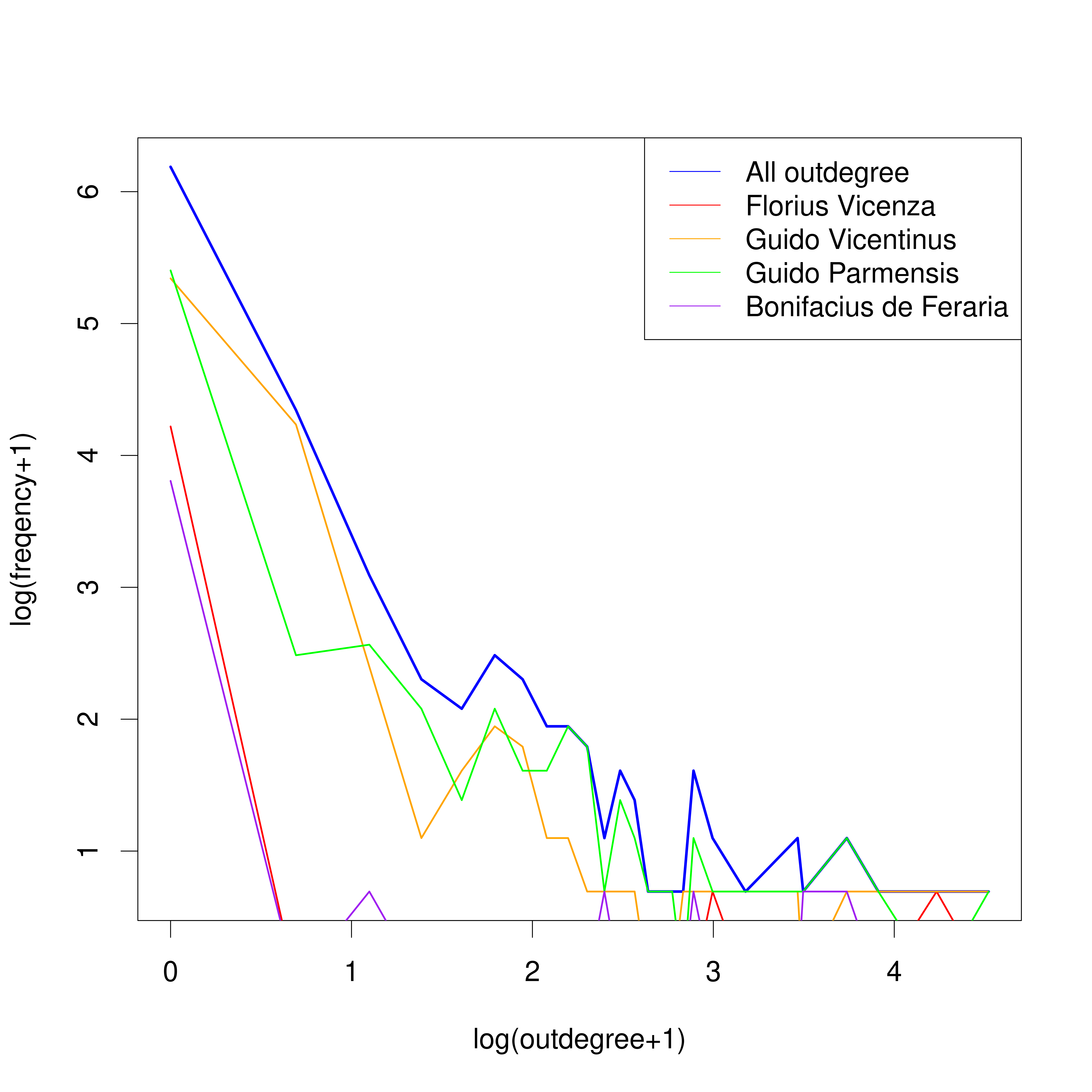

Supplement: S1 Fig — Axes are logarithmic. (TIFF) [file pone.0315467.s005.tiff]

proportion of statistics

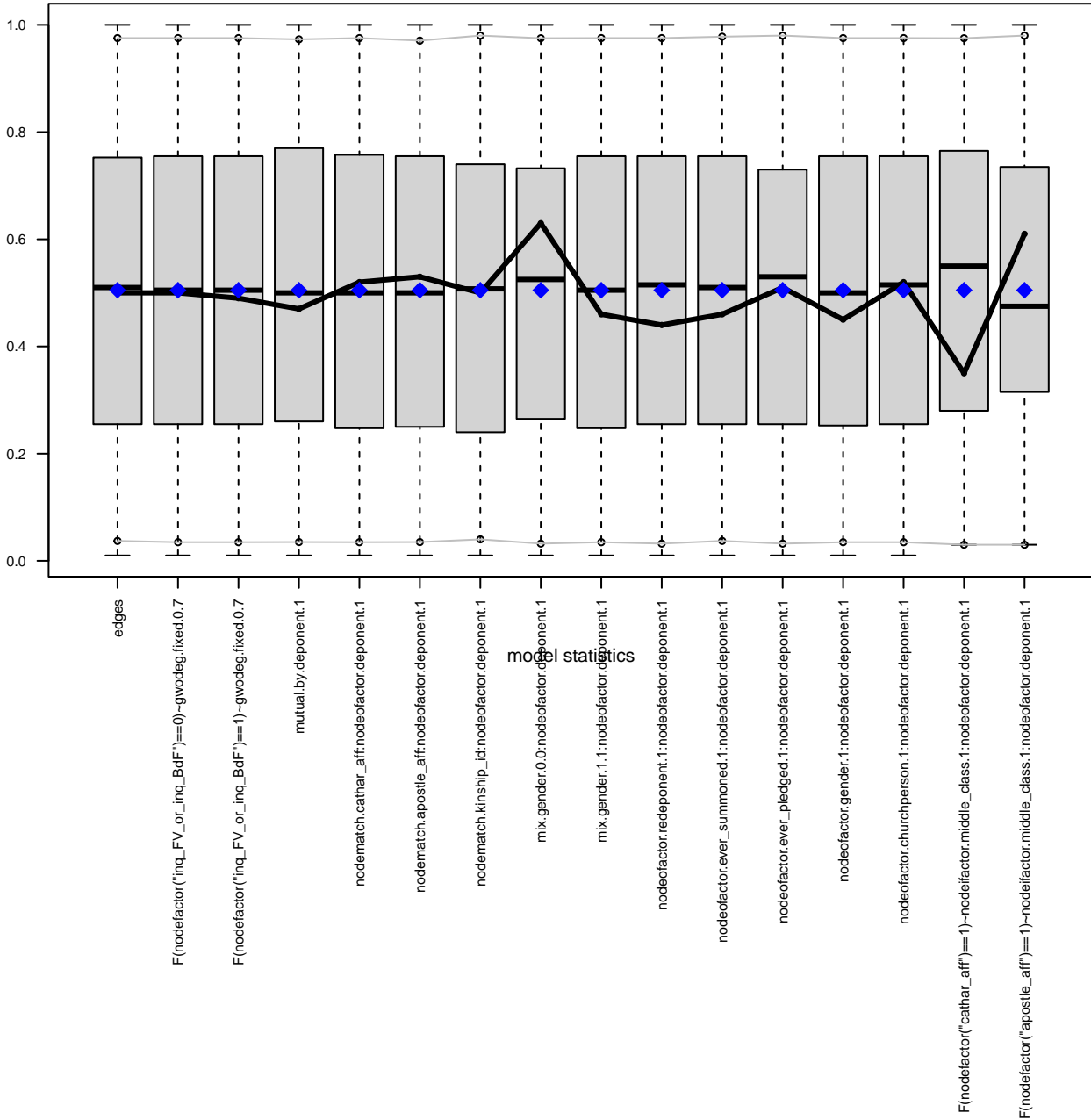

proportion of nodes

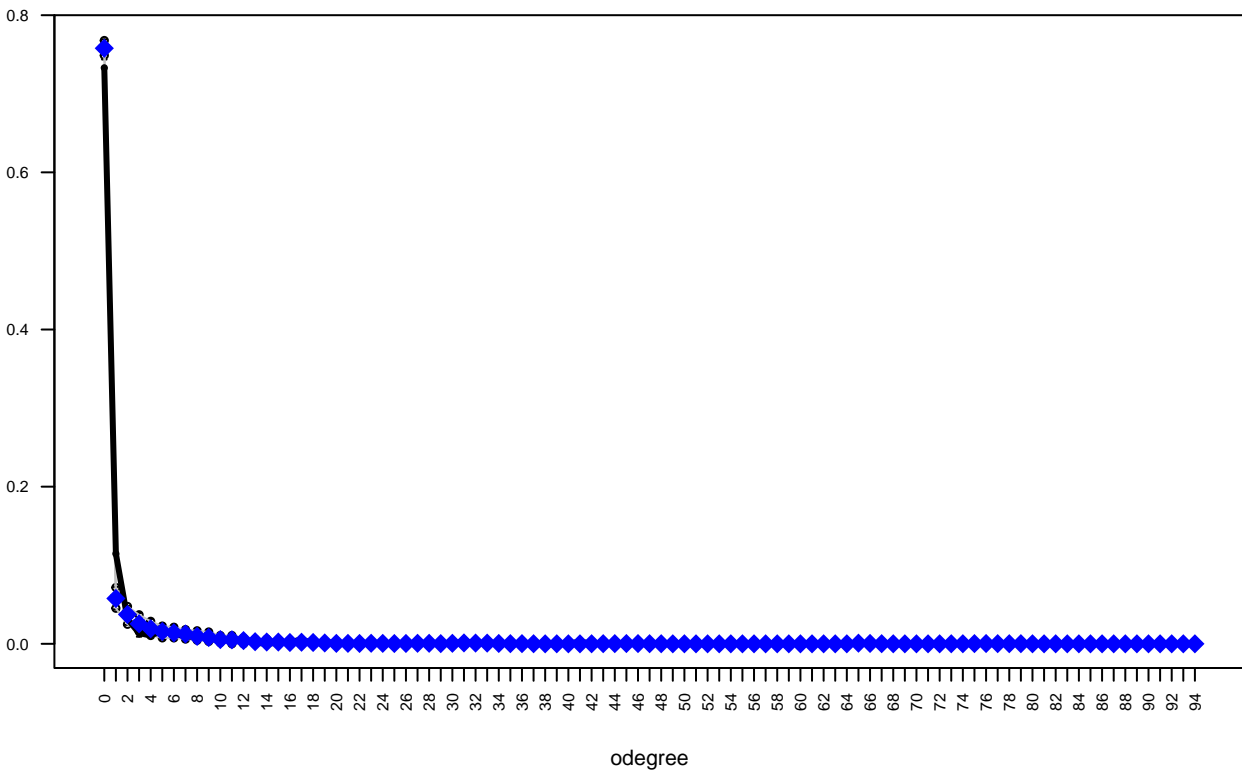

proportion of nodes

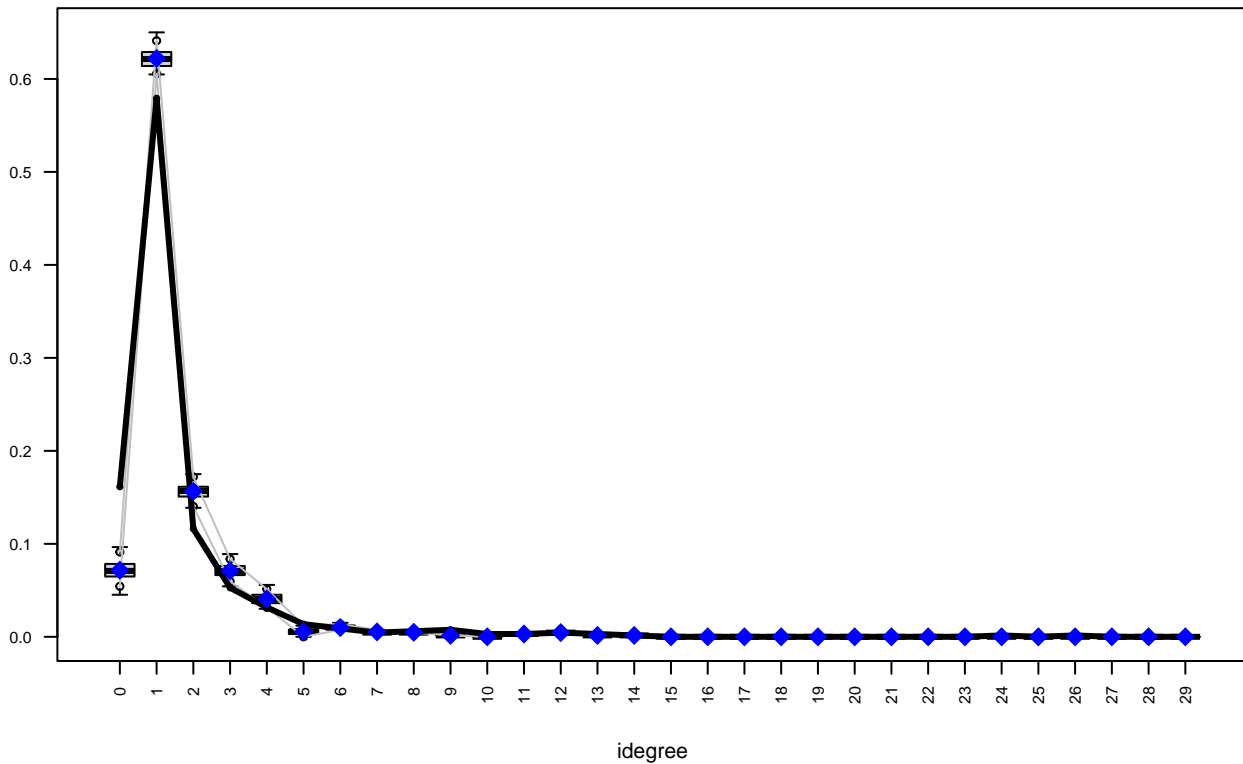

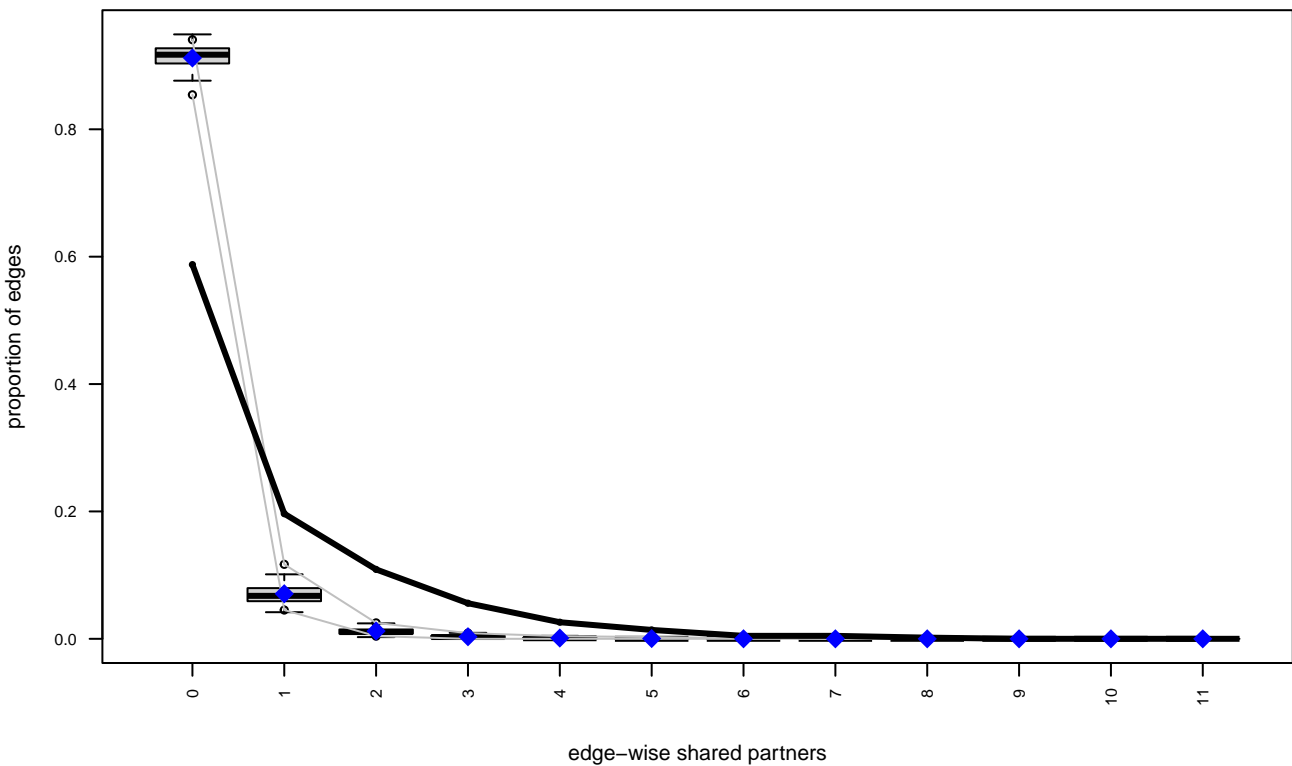

proportion of dyads

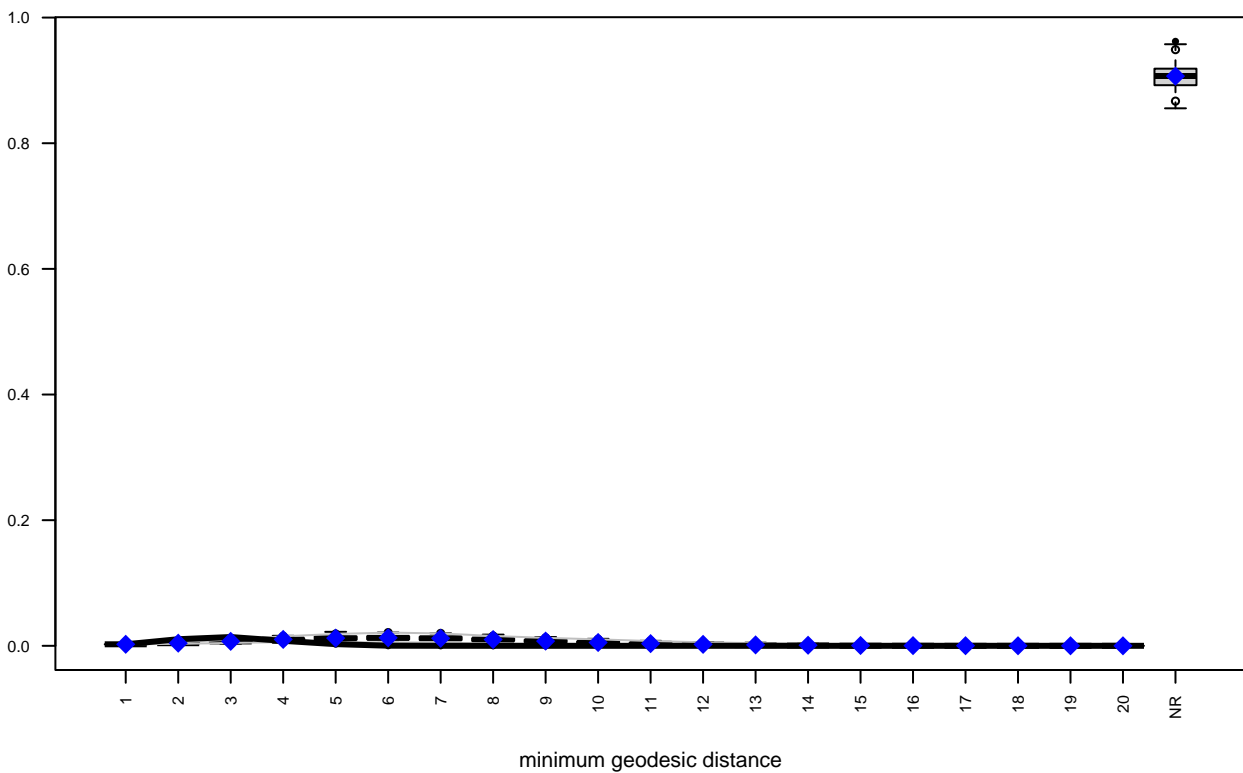

Supplement: S2 Document — (PDF) [file pone.0315467.s007.pdf]
