# Supplementary material for: Gender, kinship, and other social predictors of incrimination in the inquisition register of Bologna (1291–1310): Results from an exponential random graph model
Source: PLoS One. 2025 Feb 11;20(2):e0315467. doi: 10.1371/journal.pone.0315467 (PMC11813156; doi:10.1371/journal.pone.0315467)
Supplement: S1 Document — (PDF) [file pone.0315467.s006.pdf]

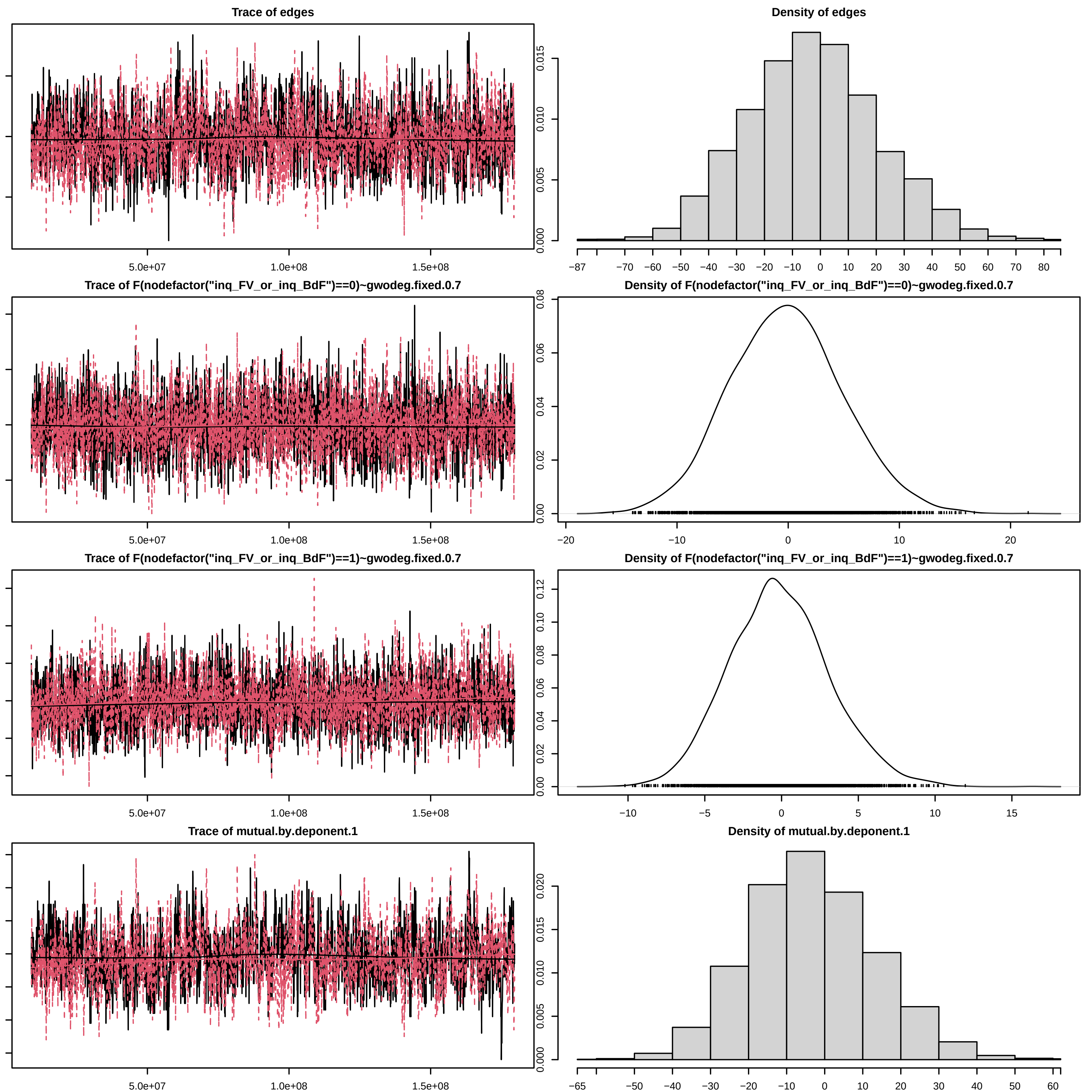
Trace of edges
5.0e+07
1.0e+08
1.5e+08
Density of edges
0.000
0.005
0.010
0.015
−87 −70 −60 −50 −40 −30 −20 −10 0 10 20 30 40 50 60 70 80
Trace of F(nodefactor("inq_FV_or_inq_BdF")==0)~gwodeg.fixed.0.7
Density of F(nodefactor("inq_FV_or_inq_BdF")==0)~gwodeg.fixed.0.7
0.00
0.02
0.04
0.06
0.08
−20 −10 0 10 20
Trace of F(nodefactor("inq_FV_or_inq_BdF")==1)~gwodeg.fixed.0.7
Density of F(nodefactor("inq_FV_or_inq_BdF")==1)~gwodeg.fixed.0.7
0.00
0.02
0.04
0.06
0.08
0.10
0.12
−10 −5 0 5 10 15
Trace of mutual.by.deponent.1
Density of mutual.by.deponent.1
0.000
0.005
0.010
0.015
0.020
−65 −50 −40 −30 −20 −10 0 10 20 30 40 50 60

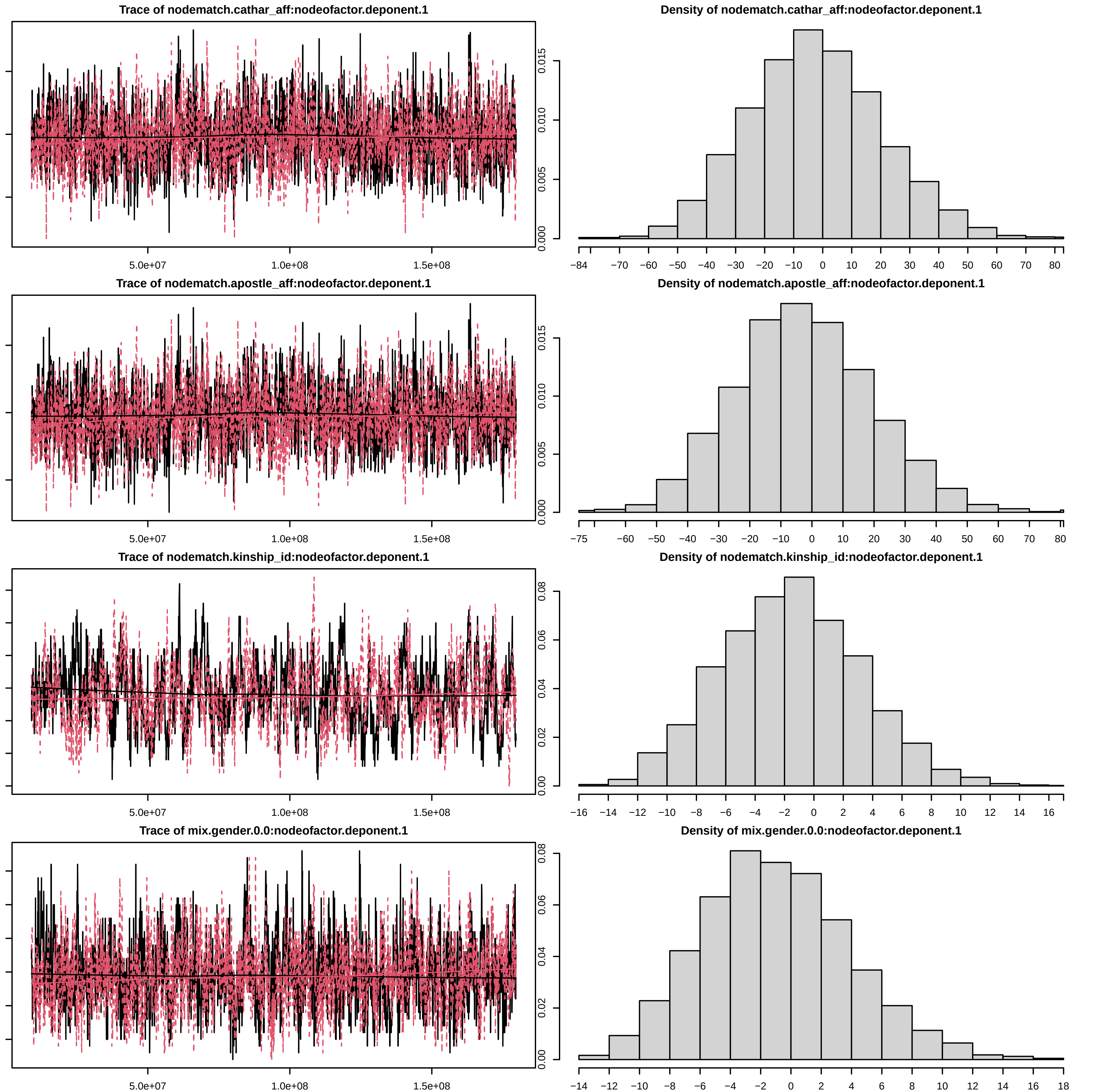
Trace of nodematch.cathar_aff:nodeofactor.deponent.1
Density of nodematch.cathar_aff:nodeofactor.deponent.1
Trace of nodematch.apostle_aff:nodeofactor.deponent.1
Density of nodematch.apostle_aff:nodeofactor.deponent.1
Trace of nodematch.kinship_id:nodeofactor.deponent.1
Density of nodematch.kinship_id:nodeofactor.deponent.1
Trace of mix.gender.0.0:nodeofactor.deponent.1
Density of mix.gender.0.0:nodeofactor.deponent.1
5.0e+07
1.0e+08
1.5e+08

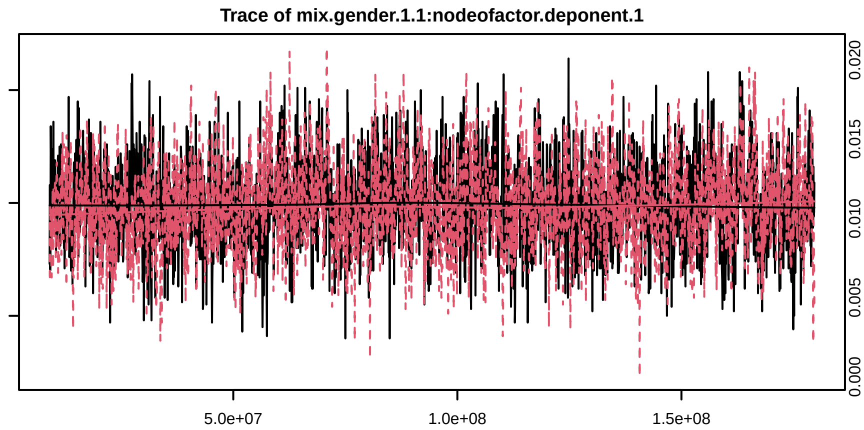
Trace of mix.gender.1.1:nodeofactor.deponent.1
5.0e+07
1.0e+08
1.5e+08

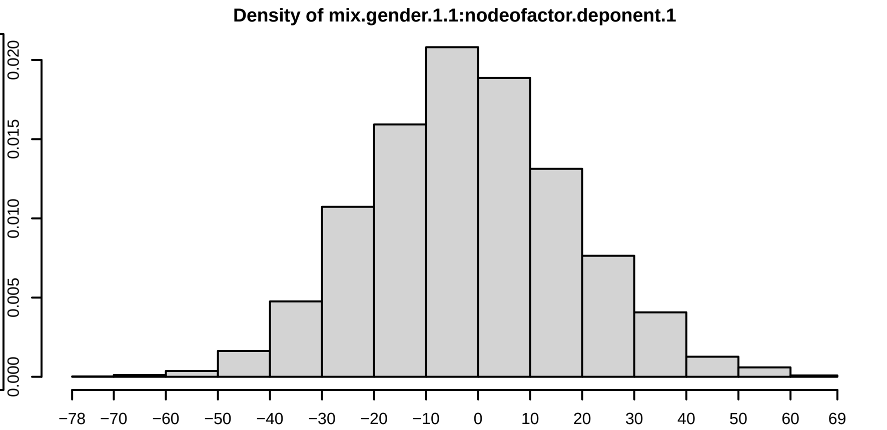
Density of mix.gender.1.1:nodeofactor.deponent.1
0.000
0.005
0.010
0.015
0.020
−78 −70 −60 −50 −40 −30 −20 −10 0 10 20 30 40 50 60 69

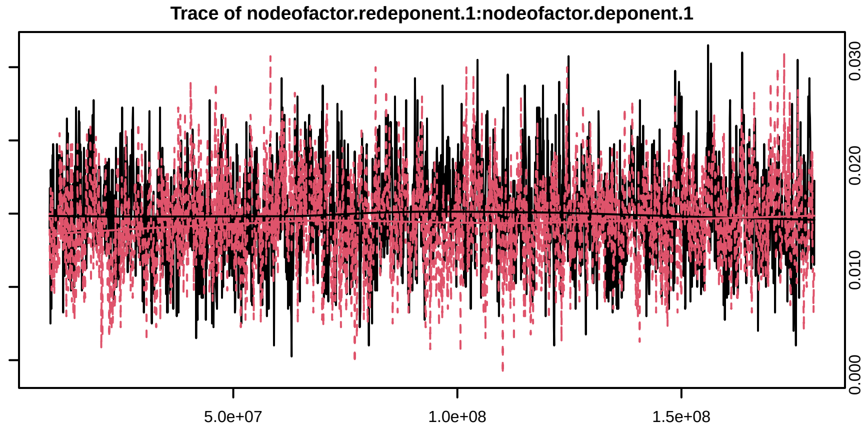
Trace of nodeofactor.redeponent.1:nodeofactor.deponent.1
5.0e+07
1.0e+08
1.5e+08

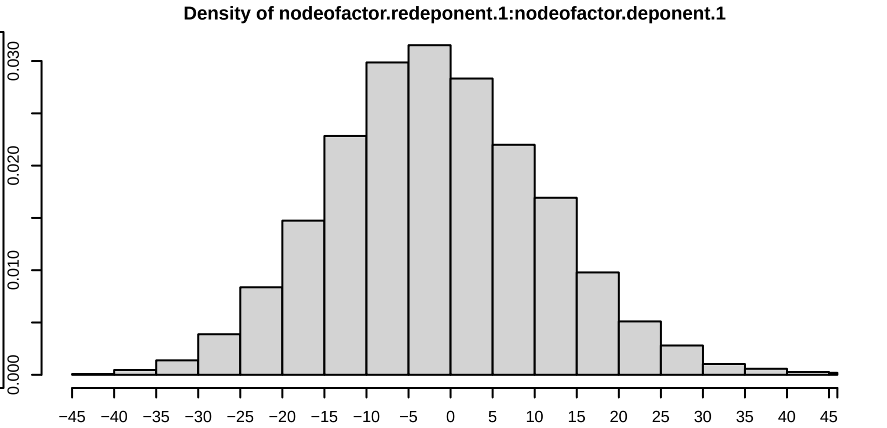
Density of nodeofactor.redeponent.1:nodeofactor.deponent.1
0.000
0.010
0.020
0.030
−45 −40 −35 −30 −25 −20 −15 −10 −5 0 5 10 15 20 25 30 35 40 45

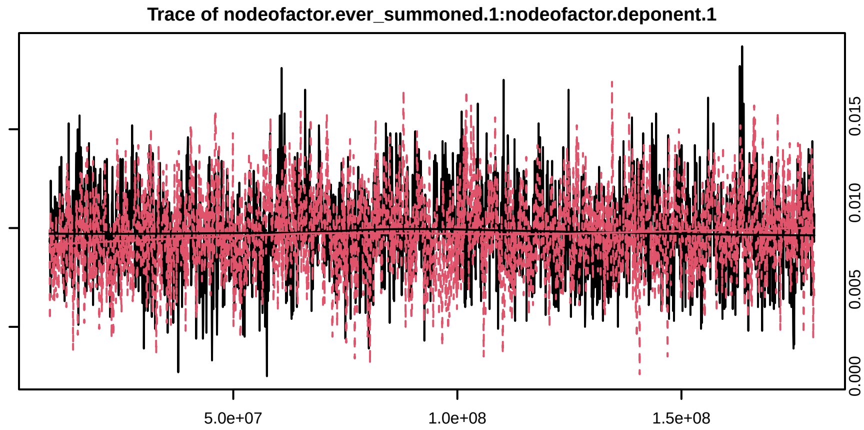
Trace of nodeofactor.ever_summoned.1:nodeofactor.deponent.1
5.0e+07
1.0e+08
1.5e+08

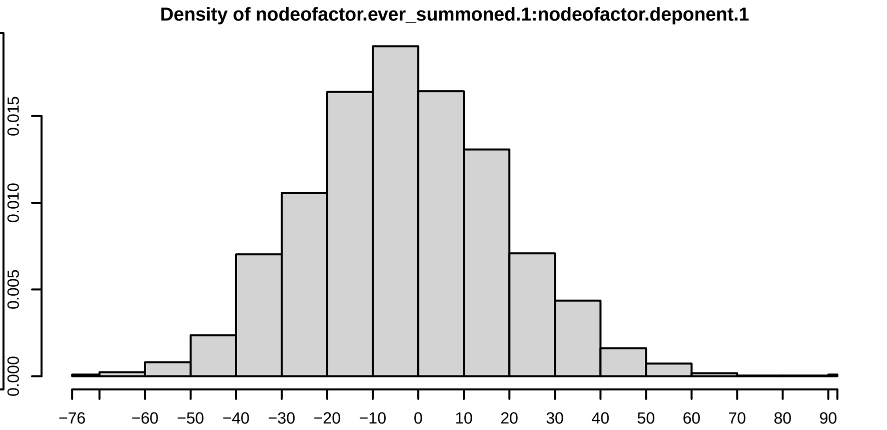
Density of nodeofactor.ever_summoned.1:nodeofactor.deponent.1
0.000
0.005
0.010
0.015
−76 −60 −50 −40 −30 −20 −10 0 10 20 30 40 50 60 70 80 90

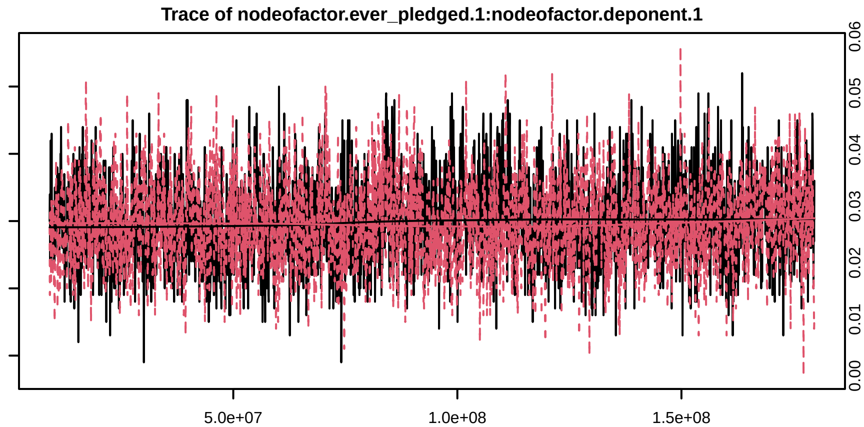
Trace of nodeofactor.ever_pledged.1:nodeofactor.deponent.1
5.0e+07
1.0e+08
1.5e+08

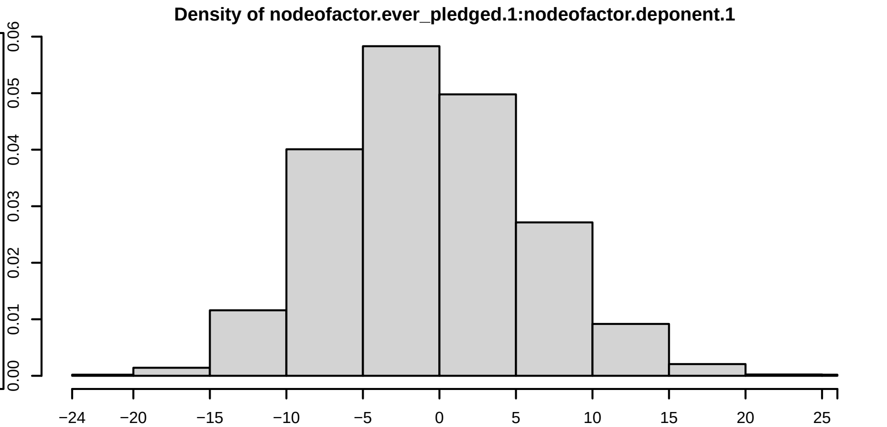
Density of nodeofactor.ever_pledged.1:nodeofactor.deponent.1
0.00
0.01
0.02
0.03
0.04
0.05
0.06
−24 −20 −15 −10 −5 0 5 10 15 20 25

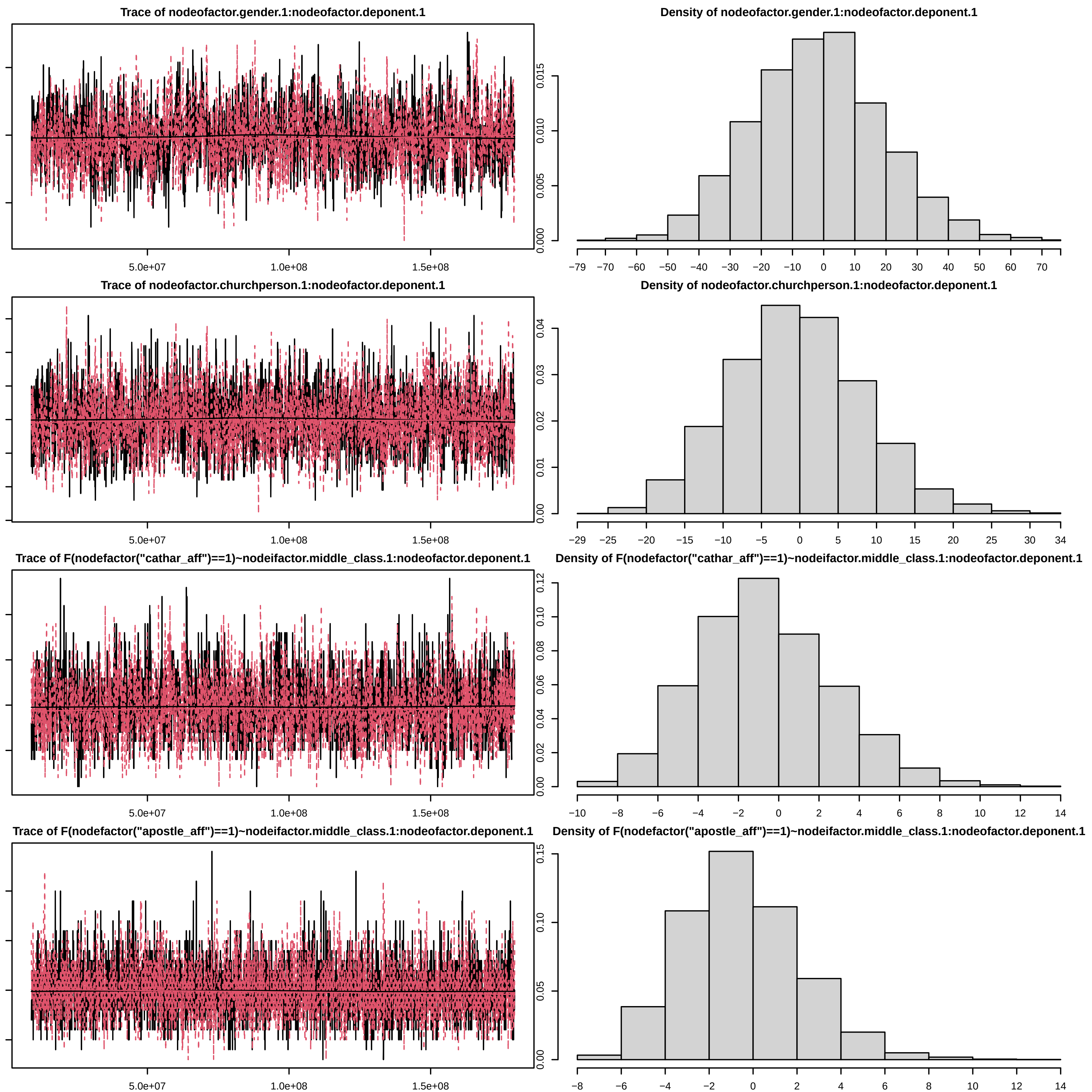
Trace of nodeofactor.gender.1:nodeofactor.deponent.1
5.0e+07
1.0e+08
1.5e+08
Density of nodeofactor.gender.1:nodeofactor.deponent.1
0.000
0.005
0.010
0.015
−79 −70 −60 −50 −40 −30 −20 −10 0 10 20 30 40 50 60 70
Trace of nodeofactor.churchperson.1:nodeofactor.deponent.1
5.0e+07
1.0e+08
1.5e+08
Density of nodeofactor.churchperson.1:nodeofactor.deponent.1
0.00
0.01
0.02
0.03
0.04
−29 −25 −20 −15 −10 −5 0 5 10 15 20 25 30 34
Trace of F(nodefactor("cathar_aff")==1)~nodeifactor.middle_class.1:nodeofactor.deponent.1
5.0e+07
1.0e+08
1.5e+08
Density of F(nodefactor("cathar_aff")==1)~nodeifactor.middle_class.1:nodeofactor.deponent.1
0.00
0.02
0.04
0.06
0.08
0.10
0.12
−10 −8 −6 −4 −2 0 2 4 6 8 10 12 14
Trace of F(nodefactor("apostle_aff")==1)~nodeifactor.middle_class.1:nodeofactor.deponent.1
5.0e+07
1.0e+08
1.5e+08
Density of F(nodefactor("apostle_aff")==1)~nodeifactor.middle_class.1:nodeofactor.deponent.1
0.00
0.05
0.10
0.15
−8 −6 −4 −2 0 2 4 6 8 10 12 14
